# Medical students’ perceptions towards implementing case-based learning in the clinical teaching and clerkship training

**DOI:** 10.1186/s12909-024-05183-x

**Published:** 2024-02-27

**Authors:** Moataz Salaheldin Gasim, Maram Hamza Ibrahim, Waad Abdelmoniem Abushama, Ikhlas Mohamed Hamed, Ibrahim Abdelrhim Ali

**Affiliations:** 1 Faculty of Medicine, The National Ribat University, Khartoum, Sudan; 2 Department of Community Medicine, Faculty of Medicine, The National Ribat University, Khartoum, Sudan; 3 Department of Physiology, Faculty of Medicine, The National Ribat University, Khartoum, Sudan

**Keywords:** Case base learning, CBL, Learning

## Abstract

**Background:**

Depending on the subject area and the ‘case’ used, many methods can be used to describe case-based learning (CBL). The majority of health professional education is patient-centered. As a result, clinical presentations and diseases are combined with social and clinical sciences, and student learning is linked to real-world applications. The purpose of this study was to evaluate how medical students at the Faculty of Medicine, National Ribat University, felt about the implementation of CBL.

**Methods:**

This descriptive cross-sectional study was conducted on 171 final-year medical students (100 females and 71 males). Students were voluntarily invited to complete a self-administered questionnaire consisting of 15 closed-ended questions with 5-point Likert scale responses, covering data on perception, awareness, and barriers to CBL.

**Results:**

The CBL satisfaction rate among medical students was 92.4%. The mean value of the medical student’s perception was 3.7 out of 5. Regarding perceptions of CBL, 65.5% of students agreed with the positive impact of CBL on their academic performance. “8.2%” (14/171) of students strongly concur that CBL improved teamwork, while “31.6%” (54/171) strongly disagree. “36.3%” of students strongly believe that CBL improved their ability to use clinical reasoning. Regarding CBL barriers, 53% of medical students considered a group of twenty participants per session to be a barrier. (69%) of students refused to consider physical presence as a barrier. “76.6%” of the students agreed that the moderator’s approach and style can have a big influence on the CBL session’s outcome.

**Conclusion:**

Overall, students had positive perceptions of CBL. Academic performance, clinical reasoning, teamwork, and information retention and retrieval were all improved by incorporating CBL into training modules. Students agreed that the group size of 20 students per session was a barrier, despite their moderate to excellent knowledge of CBL. Preparation for CBL is both time-consuming and tiring. Despite this, students agree that CBL has a positive impact on the learning process.

## Background

The medical education process has changed over the last two decades from traditional teacher-centered methods to more modern student-centered methods where students are actively involved in their learning. Clinical case-based learning (CBL) is one of the best methods for promoting student learning [1].

Thistle Thwaite et al. provide an insightful definition of CBL: “Through the use of real-world clinical scenarios, CBL aims to prepare students for clinical practice. By applying knowledge to the cases and using inquiry-based learning techniques, it builds a conceptual bridge between theory and practice [2].

Since Dr. James Lorrain Smith developed the ‘case method of teaching pathology’ while a professor at the University of Edinburgh, CBL has been widely adopted and used in the medical sciences. The method is a series of clinical-pathological correlation exercises based on the analysis of clinical cases [3, 4].

CBL improves a wide range of skills, including critical thinking, problem-solving, memory retention, and exam readiness. CBL is a cutting-edge teaching approach that has been shown to stimulate and enhance student learning. It improves students’ conceptualization, clinical reasoning, and analytical thinking. It has also helped students prepare for and perform well in clinical examinations [1].

In addition, CBL with a case-based approach gives students the freedom to discuss specific scenarios that resemble or are often examples of real-life situations [5].

CBL is a well-known pedagogical and academic approach that emphasizes case-study teaching and inquiry-based learning; as a result, it falls somewhere between organized and guided learning. Learning exercises in health professional education are often based on patient cases. As a result, student learning is linked to real-world circumstances, as the basic, social, and clinical sciences are studied about the case and linked to clinical presentations and conditions (including health and illness). Even though many arguments are given in favor of CBL as an efficient teaching and learning strategy, very little data is cited or produced to support these arguments [6].

CBL is an active learning technique similar to problem-based learning that involves small groups and focuses on solving a given problem. It stimulates active learning and produces a more fruitful outcome [7, 8]. While PBL encourages students to acquire foundational knowledge as part of the clinical case investigation, CBL is effective for students who have already acquired this knowledge [9].

Selecting and implementing a learning method is a difficult, time-consuming task that requires intensive research to demonstrate its reliability and effectiveness. CBL is a recognized and accepted approach to teaching and learning in higher education institutions around the world.

The development of a clear and valid assessment of the benefits, efficacy, and related barriers to the full implementation of CBL as a primary learning method is discouraged in Sudan due to the need for modern curriculum improvement, along with the development of scientific studies, reports, and application trials of this learning method in higher education institutions.

Addressing the barriers to the use of modern teaching techniques and their effectiveness is crucial given the ever-evolving nature of the medical profession in general and medical education in particular.

This study aimed to explore medical students’ perceptions, effectiveness, and barriers to the implementation of case-based learning in the Faculty of Medicine at National Ribat University.

## Methods

### Study design

A descriptive cross-sectional institutionalized study was conducted among the undergraduate clerkship students at the Faculty of Medicine, The National Ribat University (NRU), Sudan, from the month of January to February 28, 2023.

### Context

The National Ribat University (NRU), a 2000-year-old institution in the Burri district of Khartoum, was the setting for this research. Since its inception, the institution has grown to include 18 different faculties, 3 centers, and 2 institutions, in addition to the original 3 faculties. The Director General of the Sudanese Police Forces acts as the Vice President of the University Council, which is headed by the Secretary of the Ministry of Interior. 1,800 first-year medical students were enrolled at the NRU during the study period. In addition, 320 students enrolled in the internship component of their studies. There are currently 42 medical schools in Sudan. The NRU Faculty of Medicine and Problem-Based Learning is one of the few medical schools in Sudan that combines different teaching methods (lecture-based learning and case-based learning).

### Study population

The study population consists of undergraduate clerkship medical students who volunteered to participate and are currently enrolled at NRU in their fifth year of undergraduate studies. Written informed consent was obtained from each participant after the research procedure and objectives of the study were explained in clear, simple terms. Participants were assured that the data collected would be confidential and would only be used for research purposes. It was clearly explained that participation in this study was voluntary and that the participant had the right to withdraw at any time without any penalty. Questionnaire responses and internet data were collected anonymously by online platforms (Google forms).

#### Inclusion criteria

- Medical students in their 5th year of medical school who are undergoing their clerkship.
- Medical students at National Ribat University who have completed different medical education modalities and methodologies (lecture-based, case-based, and problem-based learning).

#### Exclusion criteria

- Pre-clerkship medical students.
- Students who did not wish to take part in the completion of the online survey.

### Sampling technique

171 medical students out of a total of 300 students (a response rate of 55.1%) agreed to participate in the total coverage sample of the fifth year of medical school.

### Data collection tools

Data were collected using a carefully pre-tested, standardized questionnaire; a pre-designed, online-based questionnaire was developed by the principal investigators. The content accuracy, reliability, and internal validity of the survey items were finalized with multidisciplinary input from the study investigators. In addition, an expert in health professions education endorsed the final structure of the questionnaire, and confirmatory factor analysis supported its validity.

The questionnaire consists of three sections with a total of 16 questions. The first section of the questionnaire tests the perceptions and knowledge of participants regarding various aspects of CBL, e.g., the definition, means, and components. The second section measures the effectiveness of CBL by questioning the benefits and skills acquired through CBL, e.g., retention and retrieval of information, teamwork, and clinical reasoning. The third section identifies the barriers and obstacles hindering the proper application of CBL among participants, e.g., the number of students per session, time and effort consumption, and the approach of the session leader.

Serial numbers were used to identify each question. Demographics (age, gender, location, and semester), perceptions, awareness, and barriers to CBL were covered in the questionnaire.

The Likert scale, which consists of the values 1 (strongly disagree), 2 (disagree), 3 (neutral), 4 (agree), and 5 (strongly agree), was used to select responses to a portion of the study questionnaire. We used the mean to determine the central tendency [10], which we used for statistical analysis and additional Likert scale inference. The percentage was used as a qualitative indicator.

A brief informed consent statement was included in the introduction to the questionnaire sent to students’ email addresses and in the opening of the online Google Form questionnaire.

### Data analysis

SPSS (Statistical Package for Social Science) version 20 was used to enter, collect, and analyze the data. Continuous data are presented as means (standard deviation) or medians (range) according to normality, while categorized variables are presented as frequencies and percentages. The Likert scale and Cronbach’s test were used to the maximum extent possible.

## Results

### Characteristics and socio-demographic details of the participants

The study involved a total of 171 medical students in their 5th year of medical school who were enrolled in undergraduate clerkships and who volunteered to take part in the study.

The study participants were divided into 71 males and 100 females, with female students making up 58.5% and male students making up 41.5% of the total study population.

At the time of the study, CBL was delivered every week for one academic semester in the 5th year.

### The awareness of medical undergraduate students in the clerkship phase with regards to CBL

**Fig. 1:**
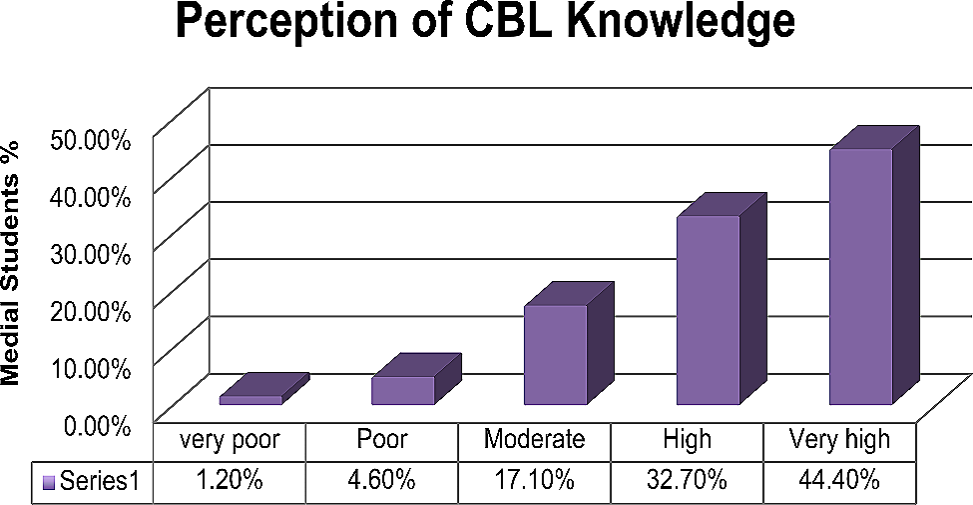
Degree of knowledge about CBL (*n* = 171)

The majority of medical students—98.2% (168/171)—reported that they were familiar with CBL, while ‘94.7%’ (162/171) of them had experience with CBL. " 77.1% (132/171) of students rated their knowledge of CBL as high to very high, as shown in Fig. 1.

When asked about the nature of CBL, “93%” (159/171) of respondents indicated that it was carried out as a group activity rather than by an individual student.

In addition, ‘92.4%’ (158/171) chose the initial topic, which is familiar to the students and for which there has been prior preparation, while ‘1.8%’ (3/171) chose the alternative.

While “84.8%” (145/171) of the medical students believed that the moderator was a student, “15.2%” (26/171) claimed that the moderator was a doctor (teacher).

The majority of medical students, or “56.7%” (97/171), are aware that the facilitator only gives short instructions. “93%” (159/171) of them are aware that using external resources and searching for data is allowed when participating in CBL sessions.

### The perception of medical undergraduate students in the clerkship phase with regards to CBL

“40.4%” (69/171) of the study participants strongly agree that CBL helped them understand the case presented, and no students disagree. Table 1.

“36.3%” (62/171) of students strongly believe that CBL has improved their ability to use clinical reasoning; “49.1%” agree and “1.2%” disagree.

In terms of evaluation, ‘8.2%’ (14/171) of students strongly agreed that CBL improved teamwork, while ‘31.6%’ (54/171) strongly disagreed.

**Fig. 2:**
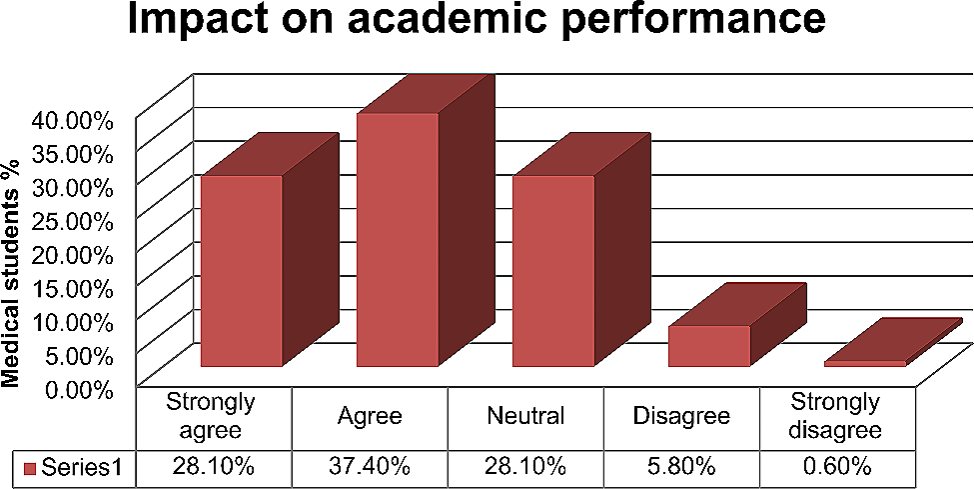
CBL impact on academic performance (*n* = 171)

In addition, ‘65.5%’ (112/171) of students agree that CBL has improved their academic performance, while ‘6.4%’ (11/171) disagree, as shown in Fig. 2.

**Table 1:** Students’ perception towards case-based learning (CBL)

Variable	Strongly disagree n (%)	Disagree n (%)	Neutral n (%)	Agree n (%)	Strongly agree n (%)	Mean Likert score ± SD / out of 5
The participation during cased based learning helped in achieving proper understanding of the presented case	0 ( 0%)	0 ( 0%)	19 (11.1%)	83 (48.5%)	69 (40.4%)	4.3 ± 0.65
CBL helped in retention and retrieval of information	0 (0%)	1 (0.6%)	23 (13.5%)	71 (41.5%)	76 (44.4%)	4.3 ± 0.71
Discussion conducted during CBL session improved clinical reasoning skills	0 ( 0%)	2 (1.2%)	23 (13.5%)	84 (49.1%)	62 (36.3%)	4.2 ± 0.71
Participation in the discussion conducted during CBL session improved team work skills	54 (31.6%)	7 (4.1%)	30 (17.5%)	66 (38.6%)	14 (8.2%)	2.9 ± 1.41
“in your opinion participation in case based learning had an active and a positive impact on your academic performance	1 (0.6%)	10 (5.8%)	48 (28.1%)	64 (37.4%)	48 (28.1%)	3.9 ± 0.91
“Do you think the style of the moderator in leading the discussion may affect the end results of CBL positively?	5 (2.9%)	9 (5.3%)	26 (15.2%)	81 (47.4%)	50 (29.2%)	2.8 ± 0.96
Total Perception Mean						3.7/5

### Barriers in the implementation of case-based learning

Four things were seen as barriers to the use of CBL. One of these was the number of students participating in the CBL session (20 students). ‘54.4%’ (93/171) of respondents agreed that this was a barrier, while ‘33.3%’ (57/171) disagreed.

When asked if physical sitting during the session was a barrier, ‘67.8%’ (116/171) of students said no, while ‘18.1%’ (31/171) said yes. This was the second barrier to the introduction of CBL.

Perceived time and effort required was the third barrier to CBL implementation; of the students surveyed, ‘62.6%’ (107/171) thought that CBL did not require much time or effort, while ‘31%’ (53/171) thought that it did.

The fourth barrier to CBL implementation was the leadership style of the team. When asked if the moderator’s approach affected the outcome of the CBL session, ‘76.6%’ (131/171) of the students agreed, indicating that the moderator’s approach and style can have a big impact, as shown in Table 2.

**Table 2:** Barriers on implementing CBL

Barrier	Yes (%)	No (%)	Neutral (%)
Number of students participating in CBL session (20 participants)	54.5%	33.3%	12.3%
Physical setting	18.1%	67.8%	14%
Time and effort spent in preparing for CBL	31%	62.6%	6.4%
Moderator’s approach affected the CBL session’s outcome	76.6%	8.2	15.2%

### Student satisfaction with CBL

**Fig. 3:**
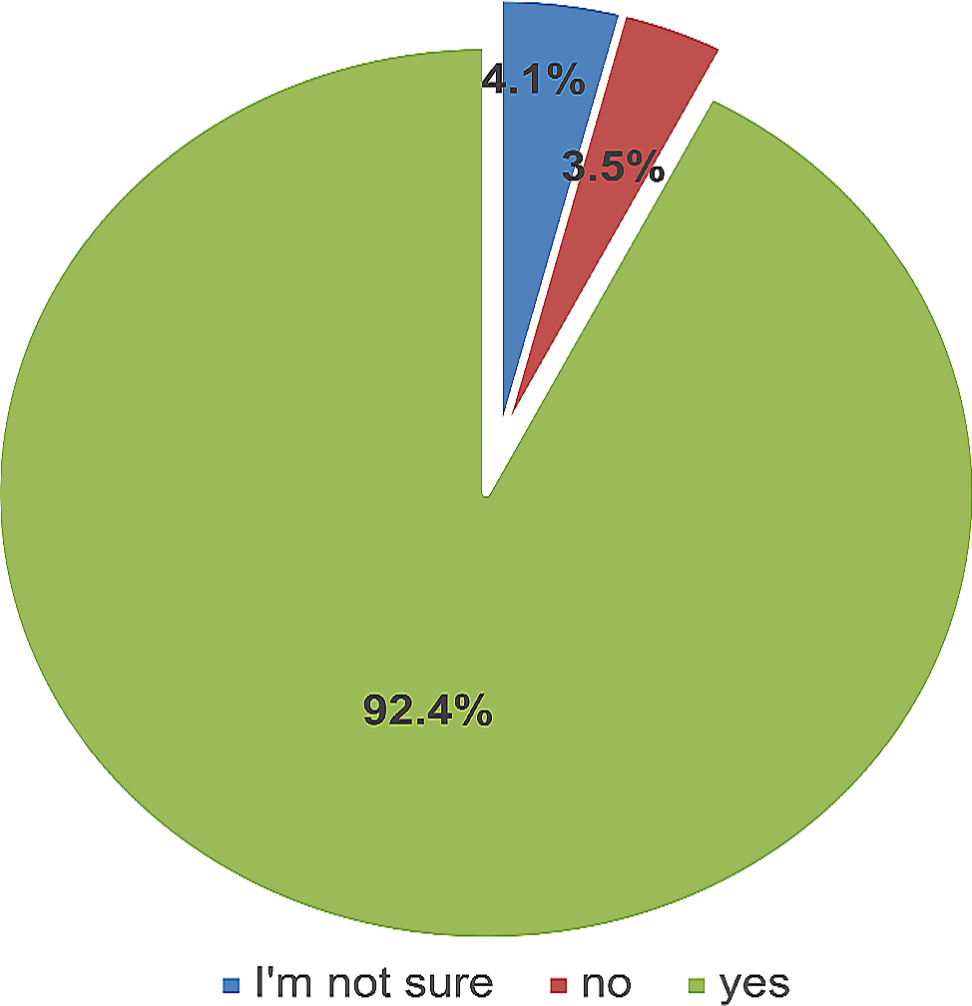
Medical student satisfaction with CBL

Most medical students (92.4%, 158/171) agreed that CBL was an effective teaching strategy. See Fig. 3.

## Discussion

In this study, a self-designed questionnaire was used to assess the benefits and challenges of implementing CBL among 5th-year medical students at the Faculty of Medicine, NRU.

Even while 37.4% and 28.1%, respectively, strongly believe that inclusion of CBL enhanced academic achievement and only 5.8% disagrees. more research showed that many students valued the program’s ability to foster critical thinking and problem-solving abilities. This is consistent with the results of previous litreature about the deeper learning capacity of CBL [11]. Nonetheless, several issues may arise regarding workload and facilitator abilities. These issues might be resolved by customizing CBL techniques according to student input and offering comprehensive facilitator training. Despite its limitations, our work adds to our knowledge of CBL’s efficacy specifically in Sudan and raises questions about how it might improve medical education. Future studies may examine the enduring effects of CBL and its flexibility in many contexts and fields of study. However, the use of CBL is an effective strategy in a study proposed in an Indian setting [12].

This study covered several aspects of CBL’s efficacy, including improving students’ ability to solve clinical problems, think analytically, and assimilate information. Also, this study confirmed that CBL encourages more participation and learning than conventional lectures. The study enhanced the idea that CBL is favoured in specific situations by instructors and students. It’s also enhancing students’ capacity to use fundamental science ideas in clinical settings.

### Impact of CBL on academic performance

In addition, this finding supports the results of a previous study that found CBL to be the most effective teaching strategy for undergraduate medical students in terms of academic performance, interest, and motivation [13]. According to another study that supports our findings, CBL pedagogy can help improve students’ academic performance while fostering a more engaging and collaborative learning environment [14]. Gurleen Kaur et al. reported no significant difference in academic performance following the implementation of CBL sessions, which is in contrast to our findings [15].

In our study the majority of students who disagreed with the statement that CBL improved academic performance did not attend the CBL sessions. According to our results, 4% of students claimed not to have attended the CBL session, whereas a higher percentage of students had been and chose to strongly agree that the CBL had a positive impact on their academic performance.

### Clinical reasoning and information retrieval

In addition, 36.3% of students strongly agreed that their participation in the discussion improved their clinical reasoning skills, and 44.4% strongly agreed that CBL helped them remember and retrieve material. In addition, 46.8% strongly agreed that the CBL training improved their teamwork skills. This is consistent with research finding that CBL improved students’ performance on MCQs [5].

### Potential effects of CBL on curriculum development and medical education

Medical education could benefit from CBL in several ways. Studies have indicated that students who finish more cases typically receive higher grades for each case [16].

Additionally, it has been discovered that case-based learning is highly beneficial in fostering more fruitful interactions between educators and learners as well as advancing students’ capacity for independent study, theory application, and self-learning.

Furthermore, it has been discovered that CBL in medical education promotes diagnostic competencies. As a result, CBL has the potential to enhance student performance, critical thinking abilities, and learning efficiency in medical education.

CBL involves giving students hypothetical or real-world problems to consider, evaluate, and resolve. By exposing students to real-world circumstances that they might face in their future employment as healthcare professionals, the use of CBL in medical education can help shape the curriculum. Students can acquire critical thinking, problem-solving, and decision-making abilities that are crucial for their professional development by examining and resolving these instances [17].

### Barriers to CBL

Depending on the situation and the technology used, there may be a variety of barriers to the implementation of CBL. However, some common barriers have been identified in the literature, such as lack of funding, technical difficulties, and lack of support [18]. However, 67.8% disagreed that the physical presence of CBL was considered a barrier or obstacle, and 31% only saw the time required to prepare for CBL as consuming and requiring a great deal of effort. In this study, the majority of participants (54.4%) did indeed agree that the number of students per session (20 participants) was a barrier to equal participation in the discussion. Therefore, we can suggest other options to optimize group size by considering the following factors: the course content, the learning objectives, the pedagogical approach, the assessment methods, and the instructor’s workload. There is no one ideal size for discussion groups, but some research suggests that smaller groups (4 or 5 students) can increase social presence, commitment, and participation [19, 20].

Also, we can enhance collaboration within larger groups by considering the following strategies: communicate your expectations and goals clearly, set an example of collaboration, use team collaboration tools, streamline complex processes, promote a community working environment, foster honest and open communication, encourage creativity, highlight individuals’ strengths, implement a team-based reward system, and improve internal communication.

In addition, 47.4% of participants in our study felt that the moderator’s style could have a good influence on the results of CBL. Add to that, there are other barriers to CBL, including, firstly, the theoretical limitations that occur when students analyze case studies; they may be limited to the theoretical aspects presented in the case, which may not fully prepare them for real-world problem-solving and decision-making. The second barrier is the challenge of contextual knowledge generation. Case-based instruction places a greater emphasis on contextually-driven knowledge generation, which can lead to uncertainties and opportunities for misunderstanding, demanding a higher level of active participation and reflection from students. Another barrier is the difficulty of implementing solutions. Students may struggle with implementing solutions to real-world problems, as case studies often focus on theoretical analysis rather than practical application.

The majority of medical students, or “92.4%” (158/171), agreed with a study that concluded that CBL can be a useful technique for improving the performance of medical students and residents and strengthening their clinical skills [12, 13]. According to a previous study, CBL improved student motivation, satisfaction and engagement. The CBL satisfaction rate among medical students was 92.4% [21].

In summary, the results of this study confirmed the previous studies that found CBL is a successful teaching strategy [12, 13], helps students do better academically [5, 14], and is one of the best ways to support student learning [1]. It can also build a conceptual bridge between theory and practice [2] and has assisted students in getting ready for and doing well on clinical exams [1]. Encourages active learning and yields more beneficial results [7, 8].

A reduction in the number of students in each session is recommended, and this can be achieved by increasing the number of classrooms, subgroups, and teachers.

### Limitations of this study

This cross-sectional study did not include a control group, pre-or post-CBL assessment, or examination. To compare and confirm the effects of CBL on academic performance, a randomized controlled trial comparing the attitudes and perceptions of two groups is recommended in order to validate these findings. Since this study only involved fifth-year medical students, more research on all clerkship students is necessary to maximize potential sources of bias and generalize the findings. Total coverage sampling includes the inability to make statistical generalizations, and limitations in generalizability due to small sample sizes and uncommon population characteristics.

## Conclusions

Incorporating CBL into modules improves clinical reasoning, teamwork, retention, and retrieval of information. Findings of this study indicate that CBL improves academic performance; however, further study with a large sample size is needed to confirm this finding. In addition, the majority of students cited the 20 participants per session as a barrier. This study recommends that CBL be incorporated into the majority of clerkship modules with a decrease in the number of students in each session, which can be accomplished by adding more teachers, classrooms, and subgroups. An alternative approach to the physical presence of CBL was recommended through distance education. Also, a study with experimental design is recommended to be conducted in order to identify the actual impact of use of CBL on student achievement.

## Data Availability

Data generated in this study are available from the corresponding author upon reasonable request with a completed Materials Transfer Agreement, excluding the materials including personally identifiable information.

## Abbreviations

CBL: Case based learning
DL: Didactic lecture
MBBS: Bachelor of Medicine and Bachelor of Surgery Degree
MCQ: Multiple-choice question
PBL: Problem based learning
SAQ: Short answer question
SPSS: Statistical Package for the Social Sciences
